# First-in-class immune-modulating small molecule Icaritin in advanced hepatocellular carcinoma: preliminary results of safety, durable survival and immune biomarkers

**DOI:** 10.1186/s12885-019-5471-1

**Published:** 2019-03-28

**Authors:** Ying Fan, Shu Li, Xiaoyan Ding, Jian Yue, Jun Jiang, Hong Zhao, Rui Hao, Weiliang Qiu, Kezhen Liu, Ying Li, Shengdian Wang, Limin Zheng, Bin Ye, Kun Meng, Binghe Xu

**Affiliations:** 1 National Cancer Center/National Clinical Research Center for Cancer/Cancer Hospital, Chinese Academy of Medical Sciences & Peking Union Medical College, Beijing, 100021 China; 2 Beijing Shenogen Biomedical Ltd, Beijing, China; 3 Beijing Ditan Hospital, Capital Medical University, Beijing, China; 4 Sinotau Pharmaceuticals Group, Beijing, China; 5 Brigham Women’s Hospital, Harvard Medical School, Boston, USA; 6 R&G PharmaStudies Co., Ltd., Shanghai, China; 7 Institute of Biophysics, Chinese Academy of Science, Beijing, China; 8 School of Life Science, Sun Yat-Sen University, Guangzhou, China

**Keywords:** Small molecule immune modulation, Phase I trial in advanced hepatocellular carcinoma

## Abstract

**Background:**

With poor prognosis and limited treatment options for advanced hepatocellular carcinoma (HCC), development of novel therapeutic agents is urgently needed. This single-arm phase I study sought to assess the safety and preliminary efficacy of icaritin in human as a potential oral immunotherapy in addition to the immune-checkpoint inhibitors.

**Methods:**

Eligible advanced HCC patients with Child-Pugh Class A or B were administered with a fixed oral dose of icaritin at either 600 or 800 mg b.i.d. The primary endpoint was safety, and the secondary endpoints included time-to-progression (TTP), overall survival (OS) and the clinical benefit rate (CBR). Icaritin treatment induced immune biomarkers and immune-modulating activities in myeloid cells were also explored.

**Results:**

No drug-related adverse events ≥ Grade 3 were observed in all 20 enrolled HCC patients. Among the 15 evaluable patients, 7 (46.7%) achieved clinical benefit, representing one partial response (PR, 6.7%) and 6 stable disease (SD, 40%). The median TTP was 141 days (range: 20-343 days), and the median OS was 192 days (range: 33-1036 days). Durable survival was observed in PR/SD patients with a median OS of 488 days (range: 72-773). TTP was significantly associated with the dynamic changes of peripheral neutrophils (*p* = 0.0067) and lymphocytes (*p* = 0.0337). Icaritin treatment induced changes in immune biomarkers-and immune-suppressive myeloid cells were observed.

**Conclusions:**

Icaritin demonstrated safety profiles and preliminary durable survival benefits in advanced HCC patients, which were correlated with its immune-modulation activities and immune biomarkers. These results suggested the potential of icaritin as a novel oral immunotherapy for advanced HCC in addition to antibody-based PD-1/PD-L1 blockade therapies.

**Trial registration:**

**Clinicaltrial.gov** **identifier.**

NCT02496949 (retrospectively registered, July 14, 2015).

**Electronic supplementary material:**

The online version of this article (10.1186/s12885-019-5471-1) contains supplementary material, which is available to authorized users.

## Background

Hepatocellular carcinoma (HCC) is the fifth most common cancer and the third leading cause of cancer death worldwide [1, 2]. More than 50% of the overall new HCC cases indeed occur in China, largely in association with chronic hepatitis B virus (HBV) infection. Although surgical resection and transplantation have improved survival of patients with small tumours, most HCC patients are inoperable due to late diagnosis at the metastatic stages. Chemotherapy in advanced HCC is generally considered as unsatisfactory with a response rate of < 10% and minimal improvement in survival. Sorafenib mono-therapy has a limited impact on HCC statistics, particularly in China and most developing countries, likely due to co-existing hepatitis B virus (HBV) infection, high cost needed and limited access to the remedy [2].

During the past decades, most of the single- or multi-targeted therapeutic phase III trials were demonstrated to be challenging to achieving acceptable objective response rate (ORR) or overall survival (OS) in advanced HCC [1, 3]. Nivolumab (CheckMate040) demonstrated promising results in advanced HCC in an early phase I/II trial [4], yet a significant fraction of HCC patients still remained not-responsive. Thus, there is a need to identify novel and cost-effective therapies aiming for survival improvement in advanced HCC patients [5].

Small molecule immune-modulating agents might be particularly suitable for treating advanced HCC patients because: 1) the dysfunctional liver is vulnerable with limited therapeutic tolerability and 2) HCC tumour microenvironment is particularly immune-tolerogenic [5]. Unfortunately, only a few small molecule-based immune therapeutic agents [6] have been explored in advanced HCC patients [7–9].

Icaritin, a single molecule with > 98% purity, is derived from *Epimedii herba*, a traditional Chinese herbal remedy used for immune modulation [10]. Icaritin treatment associated anti-cancer and immune-modulation activities through IL-6/Jak2/Stat3 pathways have been demonstrated in cancer cells [11, 12] (Additional file 1: Figure S1) as well as in immune cells including cytolytic natural killer (NK) /T cells, interferon- gamma (IFN-γ)-producing CD8+ T-cells and immune-suppressive myeloid-derived suppressor cells (MDSCs) [13]. We have previously shown that icaritin exhibits anti-proliferative activities both in cancer cells and in cancer-stem cells through the IL-6/Jak2/Stat3 pathway both in vitro and in vivo [14]*.* The present study aimed to explore the safety and immune activities of icaritin as a potential oral immunotherapy agent in advanced HCC, offering an alternative or complementary to antibody-based PD-1/PD-L1 blockade therapies.

## Methods

### Trial design and patients

Icaritin was supplied by Beijing Shenogen Biomedical Ltd. (manufactured by Kangerfu Pharmaceutical Industry Co., Beijing, China). The drug was in oral capsule form with corn oil as the main solvent vehicle (100 mg of icaritin per capsule). Based on previous toxicological data, pharmacokinetic results and a previous dose-escalating clinical phase Ia study results (Additional file 2: Figure S2), a multiple-dose trial was conducted by administering icaritin orally twice daily (b.i.d.) at two fixed doses of 600 and 800 mg employing a 28-day treatment cycle. Therapeutic activities and responses were evaluated every two treatment cycles. Treatment was continued until disease progression, intolerable toxicity, or patient’s decision on stopping the treatment. The medication was allowed to continue after confirmed disease progression at the discretion of physicians given the extremely limited choice of treatment modality in HCC. The study was performed in accordance with good clinical practices (GCPs) and the Declaration of Helsinki Guidelines. The study protocol was approved by an institutional review board (IRB), and written informed consent was obtained from all participating patients for enrolment as well as for data collection and data publication. This trial was registered in clinicaltrials.gov website (https://clinicaltrials.gov/ct2/show/NCT02496949).

### Safety and preliminary efficacy assessments

Patients were examined monthly for adverse events, including physical examination, haematological and clinical biochemical tests. Adverse events (AEs) were assessed according to the National Cancer Institute Common Terminology Criteria for Adverse Events version 4. All patients who received at least one dose of the study medication (intention-to-treat population) were assessed for safety.

Tumour assessments were performed with computed tomography or magnetic resonance imaging (MRI) at baseline and then every 2 months until confirmed disease progression. Each scan was assessed by both an investigator and a radiologist expert. Objective response was evaluated according to Response Evaluation Criteria in Solid Tumours version 1.1 (RECIST1.1) [15]. OS was measured from the date of enrolment until death from any cause. TTP was defined as the time from the date of enrolment to confirmed disease progression. Clinical benefit rate (CBR) was evaluated by calculating the percentage of subjects showing complete response (CR), partial response (PR) or stable disease (SD).

### Immune biomarkers and modulation activities in myeloid cells

The immune and haematology tests were performed in a registered clinical laboratory. The tests included platelet, neutrophil, and lymphocyte counts, which allowed for the calculation of the neutrophil-to-lymphocyte ratio (NLR), systematic immune-inflammation index (SII), and platelet-to-lymphocyte ratio (PLR) based on previously reported methods [16, 17]. The circulating biomarkers including AFP, IL-6, IL-8, IL-10, TNF-α, and IFN-γ, were measured during the time course of icaritin treatment. Immune-modulating activities of icaritin in macrophages and MDSCs in vitro were performed in bone marrow-derived macrophages and cord blood-derived MDSCs (CB-MDSCs), respectively (Fig. 3 and Additional file 3).

### Statistical analysis methods

The present study was an adaptive phase I trial that started with a dose escalation (Part I) to explore the tolerability of icaritin in different solid tumour patients followed by Part II to further evaluate the safety of icaritin at both dose levels of 600 and 800 mg b.i.d in advanced HCC (Additional file 2: Figure S2). Moreover, the preliminary drug efficacy was assessed as a secondary objective, which was not fully statistically powered with the sample size calculation. Twenty subjects may offer a high probability to observe several cases showing the preliminary activities of complete or partial response (CR, PR) and stable disease (SD) after the icaritin treatment. Icaritin was estimated to achieve a CBR of 35% in advanced HCC, and 20 subjects would provide a 75% chance to observe at least 6 cases showing clinical benefits.

Continuous variables were presented as the mean values with standard deviation, and categorical variables were presented as frequencies (percentages). The safety profile was evaluated mainly by the incidence of drug-related adverse event (AEs). Subjects demonstrating PR and SD were counted, and the percentages were calculated along with CBR. All *p* values were based on a two-sided test to define the difference, and a difference at *p* ≤ 0.05 was considered statistically significant. The OS and TTP curves were estimated by the Kaplan-Meier method along with medians and their 95% confidence intervals. A log-rank test was used to compare the survival curves between different subgroups and aimed to identify biomarkers that could differentiate the treatment effect. X-tile 3.6.1 software (Yale University, New Haven, CT, USA) was used to determine the cut-off values for the exploratory biomarker assessment [18]. All statistical analyses were performed using GraphPad Prism and SAS 9.4 (SAS, Inc., Cary NC, USA).

## Results

### Patient demographics

From November 2011 to August 2013, 20 HCC patients were enrolled in Part II as the following: enrolled first in the 600 mg b.i.d. group (*n* = 14) and then in the 800 mg b.i.d. group (*n* = 6). The patient characteristics at baseline are summarized in Table 1. The median age was 55 (range: 31–73) years old. All patients were in good performance status (ECOG 0/1), except for one patient. Most patients were categorized as Child-Pugh A (18 of 20) and BCLC stage C (19 of 20). HBV infection was predominant, and one patient had hepatitis C virus (HCV) co-infection in the 600 mg b.i.d. group. One patient in the 600 mg b.i.d. group and 3 patients in the 800 mg b.i.d. group had previously been treated with sorafenib, yet they qualified in this study based on the pre-established inclusion criteria.

**Table 1:** Baseline characteristics of HCC patients

Characteristic		Icaritin	
	600 mg bid	800mg bid	Total
	(N = 14)	(N = 6)	(*N* = 20)
Median age, years (range)	61 (31–74)	43 (33–73)	58 (31–74)
Male, no.(%)	12 (85.7)	5 (83.3)	17 (85.0)
ECOG performance status, no.(%)			
0	2 (14.3)	0 (0.0)	2 (10.0)
1	11 (78.6)	6 (100.0)	17 (85.0)
2	1 (7.1)	0 (0.0)	1 (5.0)
Macroscopic vascular invasion, no.(%)			
Yes	5 (35.7)	2 (33.3)	7 (35.0)
No	9 (64.3)	4 (66.7)	13 (65.0)
Portal vein tumor thrombus, no.(%)			
Yes	3 (21.4)	2 (33.3)	5 (25.0)
No	11 (78.6)	4 (66.7)	15 (75.0)
Bulky tumor*, no.(%)			
Yes	1 (7.1)	1 (16.7)	2 (10.0)
No	13 (92.9)	5 (83.3)	18(90.0)
Extrahepatic metastasis, no.(%)			
Yes	13 (92.9)	5 (83.3)	18 (90.0)
No	1 (7.1)	1 (16.7)	2 (10.0)
Extrahepatic metastatic site, no.(%)			
Lymph node	7 (50.0)	4 (66.7)	11 (55.0)
Lung	7 (50.0)	3 (50.0)	10 (50.0)
BCLC stage, no.(%)			
B	1 (7.1)	0 (0.0)	1 (5.0)
C	13 (92.9)	6 (100.0)	19 (95.0)
Number of tumor sites, no.(%)			
1	2 (14.3)	1 (16.7)	3 (15.0)
2	6 (42.9)	1 (16.7)	7 (35.0)
3	3 (21.4)	2 (33.3)	5 (25.0)
≥ 4	3 (21.4)	2 (33.3)	5 (25.0)
Hepatitis virus status, no.(%)			
HBV infection	12(85.7)	6 (100.0)	18 (90.0)
HCV infection	1 (7.1)	0 (0.0)	1 (5.0)
Liver cirrhosis, no.(%)			
Yes	10 (71.4)	5 (83.3)	15 (75.0)
No	4 (28.6)	1 (16.7)	5 (25.0)
Child-Pugh classification, no.(%)			
A	13 (92.9)	5 (83.3)	18 (90.0)
B	1 (7.1)	1 (16.7)	2 (10.0)
AFP > ULN (laboratory), no.(%)	13 (92.9)	6 (100.0)	19 (95.0)
Previous treatment, no.(%)			
Surgery	3 (21.4)	4 (66.7)	7 (35.0)
TACE/RFA	11 (78.6)	3 (50.0)	14 (70.0)
Sorafenib or Chemo	1 (7.1)	3 (50.0)	4 (20.0)

*Bulky tumor: Tumor mass in liver ≥10cm. Abbreviations: ECOG: Eastern Cooperative Oncology Group; BCLC: Barcelona Clinic Liver Cancer; HBV: Hepatitis B Virus; HCV: Hepatitis C Virus; ULN: Upper Limits of Normal; TACE: Transarterial Chemoembolization; RFA: Radiofrequency Ablation

### Safety and adverse events

In general, icaritin treatment was well tolerated. Of the 145 AEs reported, 115 (79.3%) were in the 600 mg b.i.d. group, and 30 (20.7%) were in the 800 mg b.i.d. group. Among the AEs, 10 (6.9%) were characterized as potentially drug-related, which included 8 AEs in the 600 mg b.i.d. group and 2 AEs in the 800 mg b.i.d. group. The most common AEs were hyperbilirubinemia (*n* = 13) followed by increased aspartate aminotransferase (*n* = 8), elevated transaminase (*n* = 7), upper respiratory infection (n = 6), and increased gamma-glutamyl transpeptidase (*n* = 5). No drug-related Grade 3/4 AEs were observed. No immune-related AEs, such as interstitial lung disease, thyroid dysfunction, or auto-immune hepatitis were found. The details of drug-related AEs are listed in Table 2.

**Table 2:** Incidence of drug-related adverse events

Drug-related AE^#^ (≥5%)	600 mg bid (N = 14)		800mg bid (N = 6)		Total (N = 20)
	Grade 1	Grade2	Grade 1	Grade 2	N (%)
Laboratory abnormality					
Leukopenia	2	1	0	0	3(15.0)
Neutropenia	2	1	0	0	3(15.0)
LDH increased	1	0	0	0	1(5.0)
Thrombocytopenia	1	0	0	0	1(5.0)
Skin and subcutaneous tissue disease					
Rash	0	1	0	0	1(5.0)
Gastrointestinal disorders					
Constipation	0	0	1	0	1(5.0)
Diarrhea	0	0	0	1	1(5.0)
Serious Adverse Event (SAE)*	600 mg bid	800 mg bid		Outcome	
Gastrointestinal bleeding	1	1		Resolved/Death	
Dyspnea	2	0		Death	
Seroperitoneum	1	0		Unresolved	
Liver abscess	1	0		Unresolved	
Cardiac sudden death	1	0		Death	
Liver function damage aggravated	0	1		Death	

^#^No grade 3/4 drug-related AEs were observed

*All SAE were judged by the investigators as drug-unrelated

Abbreviation: LDH: lactate dehydrogenase

There were eight instances of serious AEs (SAEs) among the advanced HCC patients (Table 2) as the following, which were not considered as study drug-related: 2 cases of GI bleeding, and 6 SAEs of dyspnea, haematosepsis, liver abscess, epilepsy, sudden cardiac death, and liver function failure (one for each case). These SAEs are commonly observed in advanced HCC patients with more refractory conditions, but none of the SAEs were considered drug-related per the clinical investigator’s assessments.

### Preliminary clinical activity and durable survival

Twelve of the fourteen patients in the 600 mg b.i.d. group were evaluated for best response as the following: one (8.3%) achieved PR; five (41.7%) attained SD, and six (50.0%) demonstrated progressive disease (PD). In the 800 mg b.i.d. dose group, only three cases were evaluated: one attained SD and two demonstrated PD. Five of twenty patients were not evaluated due to withdrawal or occurrence of SAEs, such as gastrointestinal bleeding prior to imaging.

The median TTP was 141 days (range: 20–343 days), and the median OS was 192 days (range: 33–1036 days). In the 600 mg b.i.d. group, 5 cases (41.7%) received icaritin for at least 5 cycles (5 months) before PD was confirmed. In the 800 mg b.i.d. group, one subject (16.7%) completed 4 cycles of treatment. Durable survival was observed in PR/SD patients with a median OS of 488 days (range: 72–773 days).

**Fig. 1:**
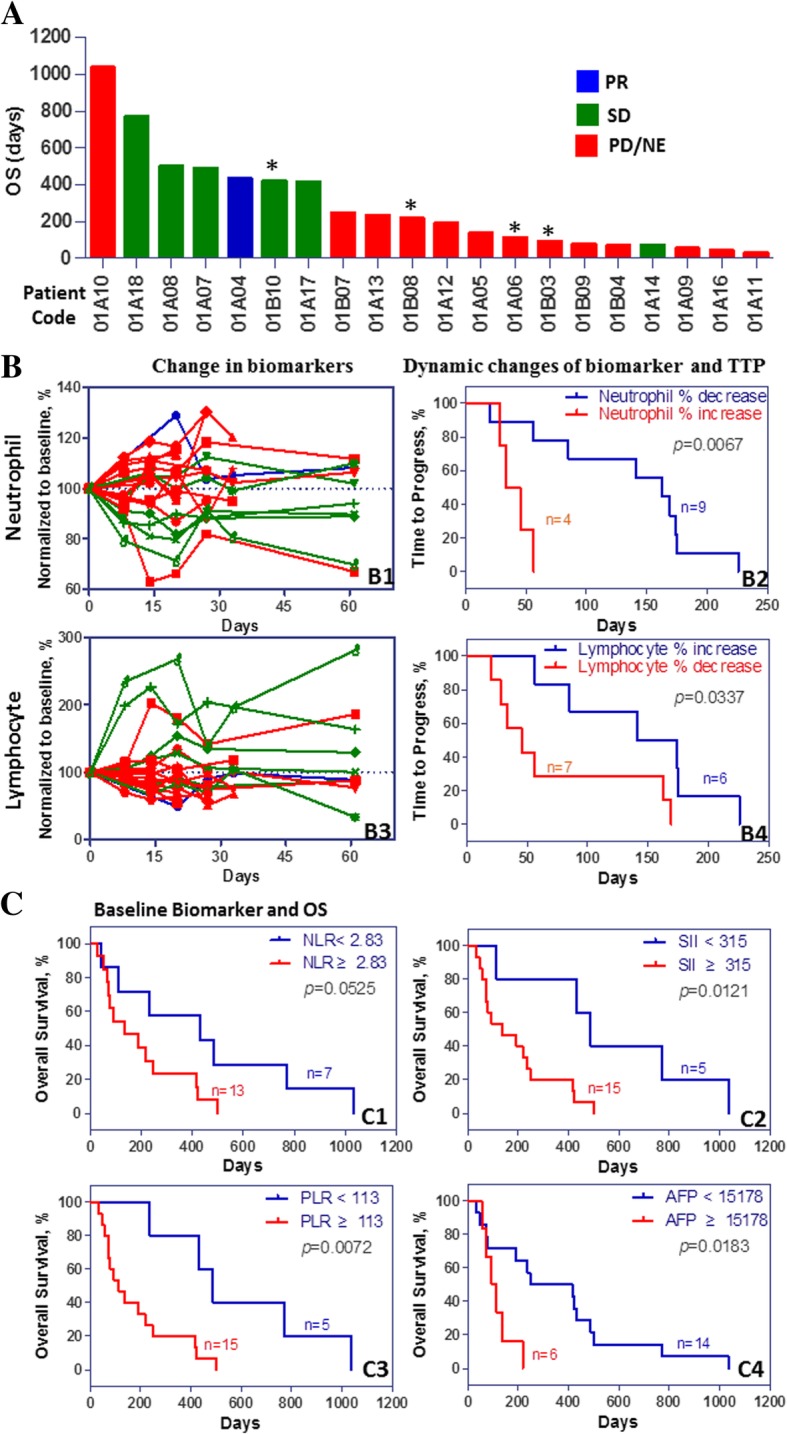
Icaritin induces anti-cancer activities in advanced HCC patients. **a**. Plot view of overall survival (OS) of all enrolled 20 (15 were evaluable) hepatocellular carcinoma (HCC) patients. Patients with star (*) indicate the second line treatment and with baseline refractory progression status after sorafenib or chemo-treatment. **b.** Icaritin treatment outcome was associated with the dynamic changes of circulating immune cells in advanced HCC patients. Icaritin treatment induced changes of neutrophil percentage were normalized with baseline as 100% (B1, green lines show stable disease (SD) patients with transient decreases in neutrophils). Icaritin treatment induced changes of neutrophil from baseline to D8 were significantly associated with time-to-progression (TTP) (p = 0.0067, B2). Dynamic changes of lymphocyte percentage were normalized to baseline as 100% (B3, green lines show SD patients with transient increases in lymphocytes after icaritin treatment). Icaritin treatment induced lymphocyte changes from baseline to D8 were significantly associated with TTP (p = 0.0337, B4). **c**. Baseline levels of inflammation and immune cell indices including neutrophil-to-lymphocyte ratio (NLR, C1), inflammation-immune index (SII, C2), and platelet-to-lymphocyte ratio (PLR, C3) and AFP (C4), were associated with OS in HCC. Median values were used as cut-off for survival estimation per the Kaplan-Meier analysis

As described above, patients were allowed to continue the study medication after tumour progression. Of the 6 patients choosing to continue (take off-study) icaritin treatment in the 600 mg b.i.d. group, five patients had an extension of more than 6 months (median: 450 days, range: 197–977 days), and one had an extension of only 88 days due to sudden death from cardiac disease (Table 2). One patient who continuously took icaritin reached the longest survival time of 1036 days (Fig. 1A).

### Immune dynamics and biomarkers

To characterize the immune dynamics associated with icaritin treatment induced survival improvement in advanced HCC, a panel of immune-inflammation dynamic indices, including neutrophils, lymphocytes and platelets, along with NLR, SII and PLR were explored. With one-year survival as the cut-off, two clinical-benefit subgroups were identified as the following: 7 patients (35%) with a median OS of 488 days and 13 patients (most are refractory with less than 2 treatment cycles) with a median OS of 95 days (Fig. 1A). The decrease in neutrophils and increase in lymphocytes were significantly (*p* = 0.0067 and *p* = 0.0337, respectively) associated with TTP by Kaplan-Meier analysis (Fig. 1B).

The OS was also significantly associated with baseline biomarkers using the cut-off values of 2.83 for NLR (*p* = 0.0525), 315 for SII (*p* = 0.0121), 113 for PLR (*p* = 0.0072) and 15,178 for AFP (*p* = 0.0183) (Fig. 1C). A significant difference between the HCC subgroups of OS≥365 vs. OS< 365 days with baseline PLR medians of 80.25 vs. 177.51 (*p* = 0.0395, Additional file 4: Figure S4) was observed. These observations were consistent with the previous finding that PLR may predict HCC treatment outcomes [19] and immunotherapeutic efficacy [20]. Interestingly, after icaritin treatment, circulating plasma levels of IL-6, IL-7, IL-8, IL-10, IL-15, AFP, and DKK1 were decreased by up to 2 folds, and circulating plasma levels of IFN-γ were increased by up to 3 folds in PR and SD, but not in PD patients (Additional file 5: Figure S5).

### Case report

**Fig. 2:**
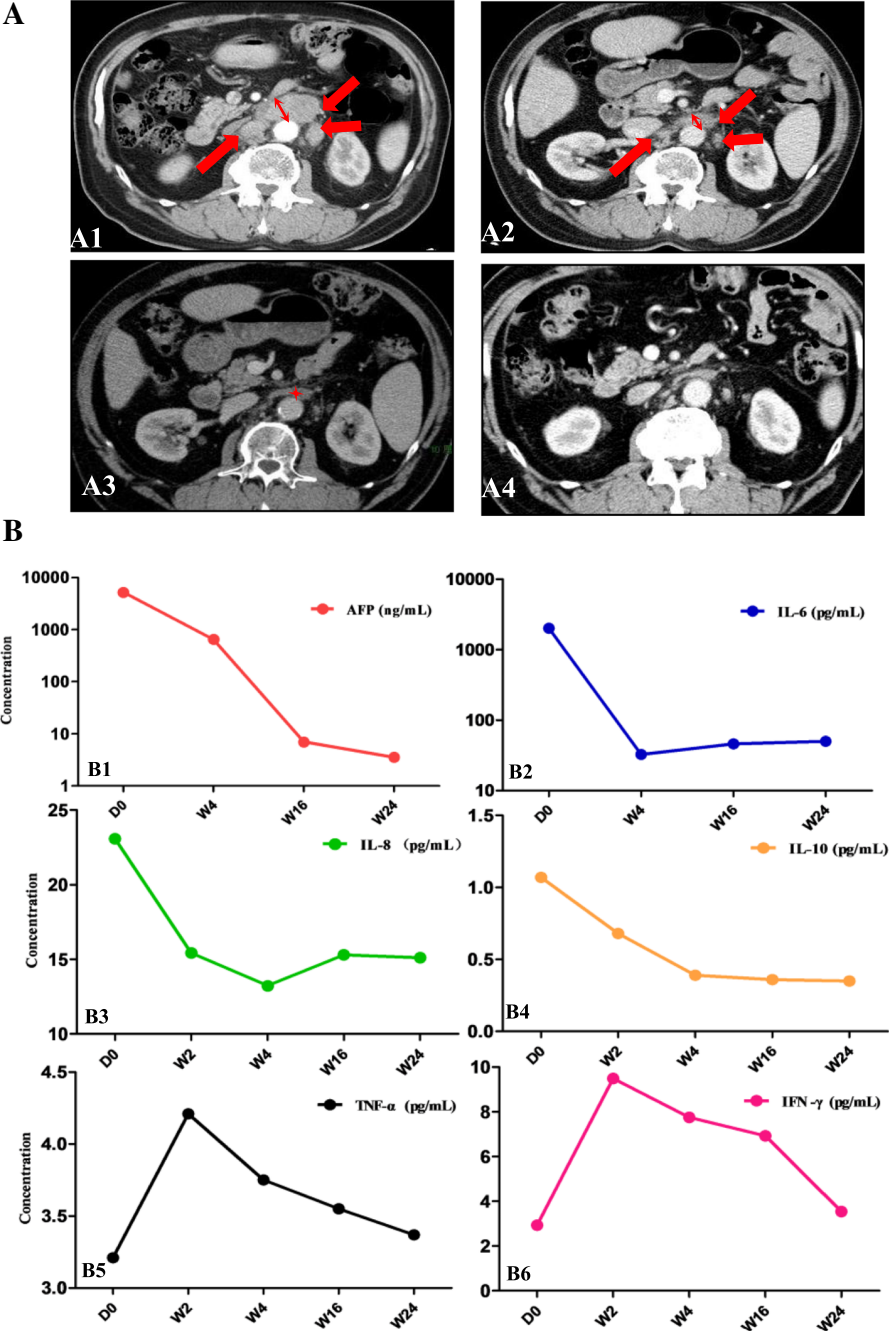
Icaritin induces durable partial response and immune biomarkers. **a.** Time course images (MRI and RECIST1.1) of a PR patient demonstrate tumour shrinkage after 8, 16 and 32 weeks of icaritin treatment compared to the baseline image. The CT images show that the retroperitoneal multiple lymph node metastases (not liver tumour lesion; multiple lesion sites are indicated by the arrows in red) were significantly reduced during the time course of icaritin treatment. The red arrows indicate lesions with diameter > 1.5 cm at baseline and week 8 of treatment. After 16 weeks of treatment, target lesions were evaluated as complete response (CR) (with maximum measurable diameter < 0.5 cm) according to RECIST1.1. **b**. Dynamic changes of AFP biomarkers and cytokine panel (IL-6, IL-8 IL-10, ΤΝF-α, and IFN-γ) in the time course of icaritin treatment. (*Note:* The time course tumour CT 3D-tomography images may not from identical sections, but they are valid for comparison of the dynamic changes and drug efficacy. **a**) By selecting the section with the maximum diameter of lesion using the RECIST1.1 global standard, the section location reference was according to the great vessels of the retroperitoneum, especially the large vessels of the retroperitoneum. **b**) Tumour shrinkage asymmetrically. **c**) CT section of images of organs may vary from time to time due to breath and other uncontrolled factors of patient)

One patient, a 62-year-old male, had a PR after two consecutive cycles of icaritin treatment. This patient started the medication after progression on transcatheter arterial chemoembolization (TACE) due to newly identified multiple abdominal lymph node metastases. Tumour lesion shrinkage was observed at 8, 16, and 32 weeks post-treatment compared to the CT image at baseline (D0) with the RECIST1.1 evaluation standard (Fig. 2A). After 4 cycles (16 weeks) of treatment, the target lesions were assessed as CR (Fig. 2A, left bottom), and the total objective response was PR, given the presence of non-target liver lesions (Fig. 2). Baseline levels of NLR, PLR and SII were 1.66, 58, and 192, respectively. The baseline AFP level was 5216 ng/ml at pre-screening and it continuously declined to 6.7 ng/ml during treatment (Fig. 2B). Circulating IL-6, IL-8, and IL-10 levels were concomitantly decreased along with tumour shrinkage after 24 weeks of treatment. Interestingly, the IFN-γ level was increased by up to 3 folds after 2 weeks of icaritin treatment, which may indicate the cytotoxic immune T-cell priming or activation (Fig. 2B).

### Modulating immune-suppression activities in myeloid cells in vitro

**Fig. 3:**
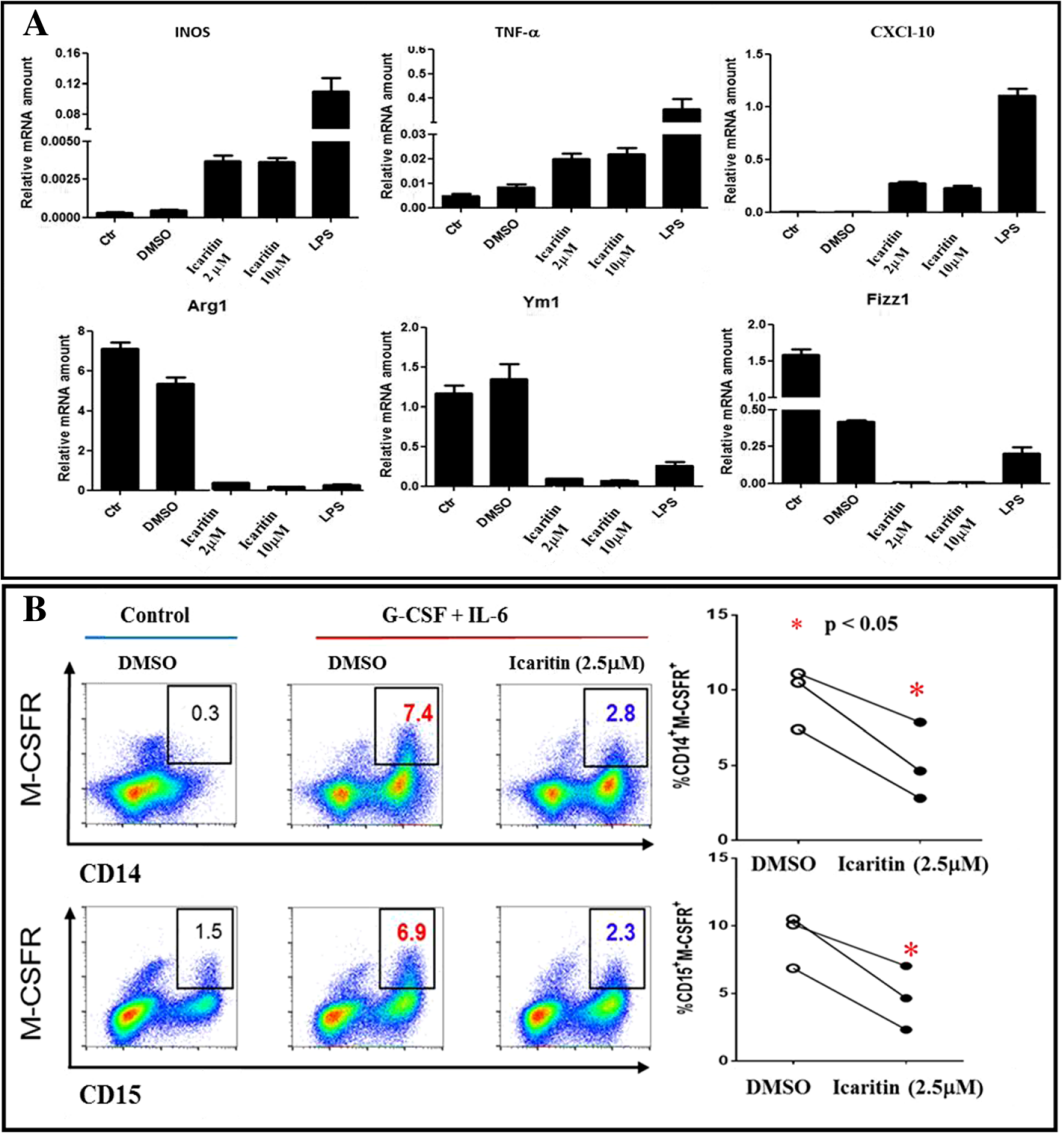
Icaritin blocks the immune-suppression in myeloid cells in vitro. **a**. Icaritin treatment induced gene expression patterns of M1-type and M2-type macrophages in vitro. The relative copy numbers of M1-type genes (INOS, TNF-α and CXCL10) and M2-type genes (Arg1, Ym1 and Fizz1) were induced by icaritin (2 and 10 μM) and normalized to β-actin. **b.** Icaritin treatment down-regulates immune MDSCs (M-CSFR expression, a key marker of activated MDSCs). Flow cytometric analysis of M-CSFR in cytokine-induced CB-MDSCs with or without icaritin (2.5 μM, 72 h) treatment with CD14^+^ and CD15^+^ sorting. Summary of cell population ratio of CD14^+^M-CSFR^+^ and CD15^+^M-CSFR^+^ cells in cytokine-induced CB-MDSCs with or without icaritin treatment (*p < 0.05, see additional file for method details)

Based on the findings that icaritin treatment-induced responses of neutrophils and lymphocytes were significantly associated with TTP, the immune-modulation activities of icaritin were investigated. After treatment of macrophages with 2 and 10 μM icaritin for 24 h, the expression of the gene panel representing M1-type macrophages (i.e., INOS, TNF-α and CXCL-10) was increased, whereas the expression of the gene panel representing M2-type macrophages was decreased (i.e., Arg-1, Ym1 and Fizz1) (Fig. 3A) [21]. In addition, the MDSC populations (both CD14^+^/M-CSFR^+^ and CD15^+^/M-CSFR^+^ cells) were significantly reduced by icaritin (2.5 μM, 72 h, *p* < 0.05) treatment (Fig. 3B). These findings may further confirm the immune-modulating associated antitumor activities of icaritin on the immune cells including MDSCs [13], neutrophils [22], and macrophages [23].

## Discussion

The main purpose of this study was to evaluate the clinical safety profiles of icaritin in advanced HCC with optimized doses. Icaritin demonstrated high tolerability (only grade 1/2 drug-related AE observed) in advanced HCC patients. The most common AE was hyperbilirubinemia, which may understandingly be disease-related. No immune-related AEs, such as interstitial lung disease, thyroid dysfunction or auto-immune hepatitis were found. Combined with data from the Part 1 dose escalation study, icaritin demonstrated favourable safety and tolerability in advanced HCC patients. Our preliminary data also showed that the response and mortalities did not correlated with icaritin dosages of 600 vs. 800 mg b.i.d. (Additional file 6: Table S1 and Additional file 7: Figure S3), which was consistent with the observations in other immune oncology trials [24, 25].

**Fig. 4:**
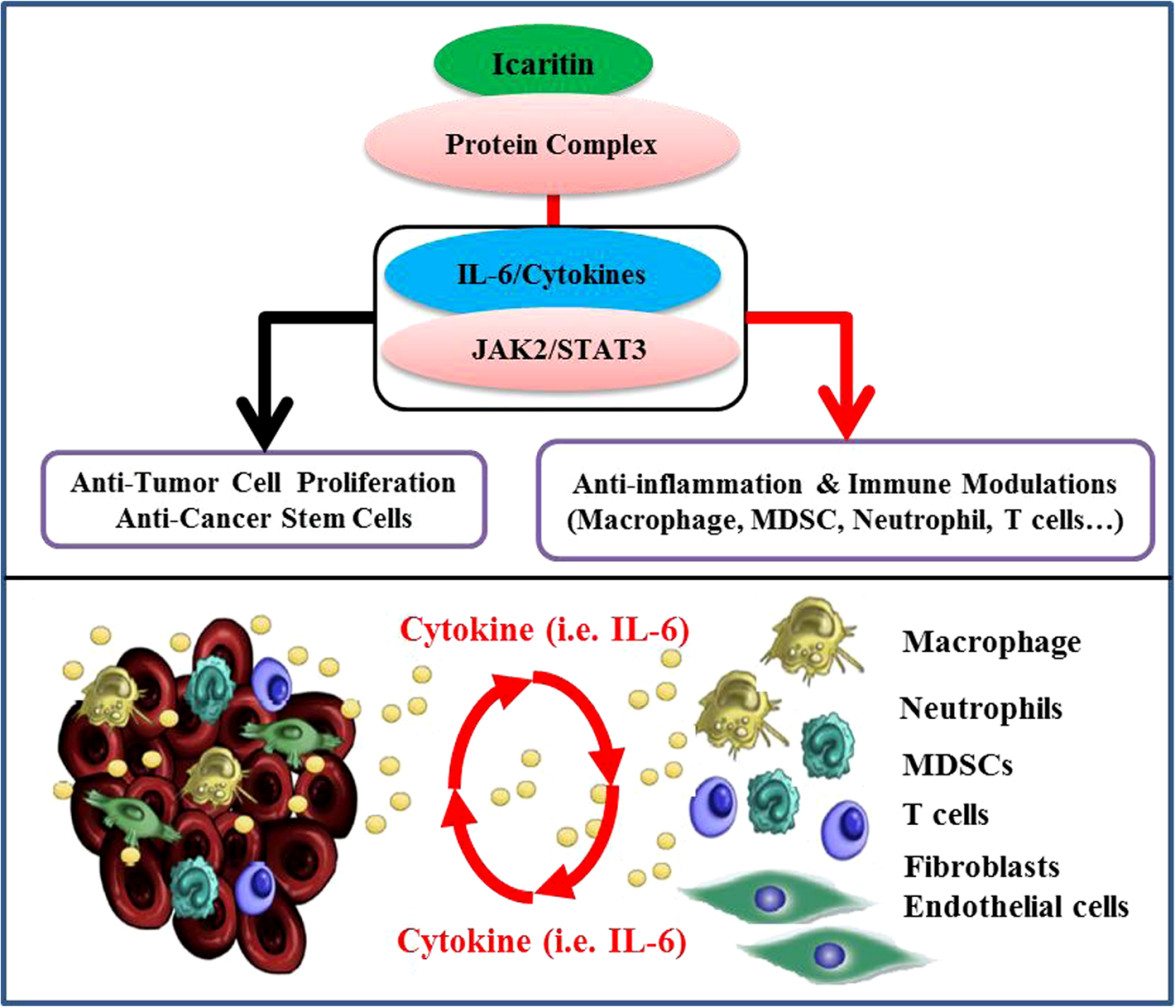
Schematic mechanism of Icaritin treatment induced anti-cancer and immune-modulation activities via IL-6/JAK2/Stat3-associated protein networking in hepatocellular carcinoma (HCC). **a.** Icaritin induced anti-tumour cell proliferation, anti-inflammation and immune modulation activities. **b.** Simplified sketch for the potential cytokine-mediated network interactions between tumour cells and immune cells in the tumour microenvironment (modified from Dr. Fisher DT et al. Semin Immunol. 2014; 26(1): 38–47. doi:10.1016/j.smim.2014.01.008)

Icaritin demonstrated preliminary efficacy of a comparable overall response rate but likely with a more durable survival compared to HCC patients treated with sorafenib in ORIENTAL trial [26] (Additional file 8: Table S2). It is well-acknowledged that the duration of cancer control and OS are critical endpoints for advanced HCC [3, 27] rather than objective response rate (ORR < 10% often observed in HCC trials and different from other solid tumours) [15, 28]. Moreover, two major confounding factors should be taken into consideration in preliminary efficacy assessment justification. First, patients enrolled in this study were much more refractory than those in the sorafenib (ORIENTAL) trial in terms of extrahepatic metastases (90% in this study vs. 68.7% in sorafenib ORIENTAL) and Child-Pugh class B (10% in this study vs. 3% in sorafenib ORIENTAL) (Additional file 8: Table S2). Second, fewer (6/20) patients were maintained on treatment after disease progression compared to that of the ORIENTAL trial, in which most patients continued with sorafenib treatment after progression. Indeed, five of the six patients had a median icaritin treatment duration of 15 months (range: 6.5–33 months), and all six patients achieved median survival of 488 days (95% CI: 406–570 days). These results suggested that post-progression treatment with icaritin may provide a durable survival benefit in advanced HCC, which was often observed in other immune oncology studies [29, 30]. In addition, the present study demonstrated that icaritin treatment significantly blocked immune-suppressive activities in myeloid cells (Fig. 3). Our results were consistent with recent findings that immune suppressive neutrophils and macrophages might be essential immune-modulating targets beyond immune checkpoint blockage (PD-1/PD-L1) [31–33]. Significance of icaritin induced anti-cancer associated immune-suppression modulation was clearly demonstrated by showing the dynamic changes in neutrophils (Fig. 1), gene expression pattern in macrophages and functional profiles in myeloid cell populations (Fig. 3). Combined data suggests that icaritin could play significant roles and contribute to the synergized anti-inflammation and anti-immune tolerance activities in advanced HCC (Fig. 4). In addition to the immune checkpoint pathways [34], modulation of immune suppressive MDSC cells and inflammation associated cytokine and chemokine may enhance the anticancer efficacy of immune therapy and patient overall survival [35–37], particularly in advanced HCC [22, 38].

## Conclusions

Anti-IL-6/Stat3 and associated molecular pathways have shown great potential in anti-inflammation and cancer immunotherapy [39–41]. Small molecule based immune-modulation and associated IL-6/STAT3 pathways deserve more translational and clinical investigations. Immune-modulation therapy clinical trial of small molecule icaritin in advanced HCC would provide valuable - supporting evidence that anti-cancer and immune-modulation activities via IL-6/Stat3-associated protein networking may help to address the challenge of high immune-tolerance in HCC, in addition to the immune checkpoint pathways (Fig. 4). The present study demonstrated preliminary clinical activities of icaritin including safety, durable survival and panel of immune biomarkers associated with anti- immune-suppression activities in myeloid cells [19, 23, 42]. Both clinical safety and preliminary efficacy of icaritin demonstrated in this report should support further clinical development in advanced HCC and other solid tumours.

## Additional files

Additional file 1: **Figure S1.** Potential molecular basis of anti-cancer and immune-modulation targets/pathways associated with icaritin and its derivatives. (PDF 179 kb)

Additional file 2: **Figure S2.** Diagram of adaptive phase I trial design. (PDF 113 kb)

Additional file 3: Supplement methods for Fig. 3. (PDF 412 kb)

Additional file 4: **Figure S4.** Correlation analysis of overall survival (OS) association with immune dynamics and biomarkers. (PDF 154 kb)

Additional file 5: **Figure S5.** Icaritin induced clinical activity and dynamic biomarkers. (PDF 181 kb)

Additional file 6: **Table S1.** Association between Dosage and Response. (PDF 248 kb)

Additional file 7: **Figure S3.** Association between dosage and mortality. (PDF 106 kb)

Additional file 8: **Table S2.** Baseline Characteristics and survival of HCC Patients (Comparison with the Chinese patients of Oriental Trial). (PDF 277 kb)

## Abbreviations

AE: Adverse events
AFP: Serum alpha-fetoprotein
CBR: Clinical benefit rate
CNS: Central nervous system
DKK1: Dickkopf-1
ECOG PS: Eastern Cooperative Oncology Group Performance Status
HBV: Hepatitis B virus
HCC: Hepatocellular carcinoma
HCV: Hepatitis C virus
IFN-γ: Interferon gamma
ITT: Intention-to-treat
MDSC: Myeloid-derived suppressor cells
NK cells: Natural killer cells
NLR: Neutrophil-to-lymphocyte ratio
OS: Overall survival
PD: Progressive disease
PLR: Platelet-to-lymphocyte ratio
PR: Partial response
RECIST: Response Evaluation Criteria in Solid Tumours
SD: Stable disease
SII: Systematic immune-inflammation index
TACE: Transcatheter arterial chemoembolization
TTP: Time to progression
ULN: Upper limit of normal
