# Supplementary material for: First-in-class immune-modulating small molecule Icaritin in advanced hepatocellular carcinoma: preliminary results of safety, durable survival and immune biomarkers
Source: BMC Cancer. 2019 Mar 28;19:279. doi: 10.1186/s12885-019-5471-1 (PMC6437929; doi:10.1186/s12885-019-5471-1)
Supplement: Supplementary file 1 — Figure S1. Potential molecular basis of anti-cancer and immune-modulation targets/pathways associated with icaritin and its derivatives. (PDF 179 kb) [file 12885_2019_5471_MOESM1_ESM.pdf]

Fig. 1S

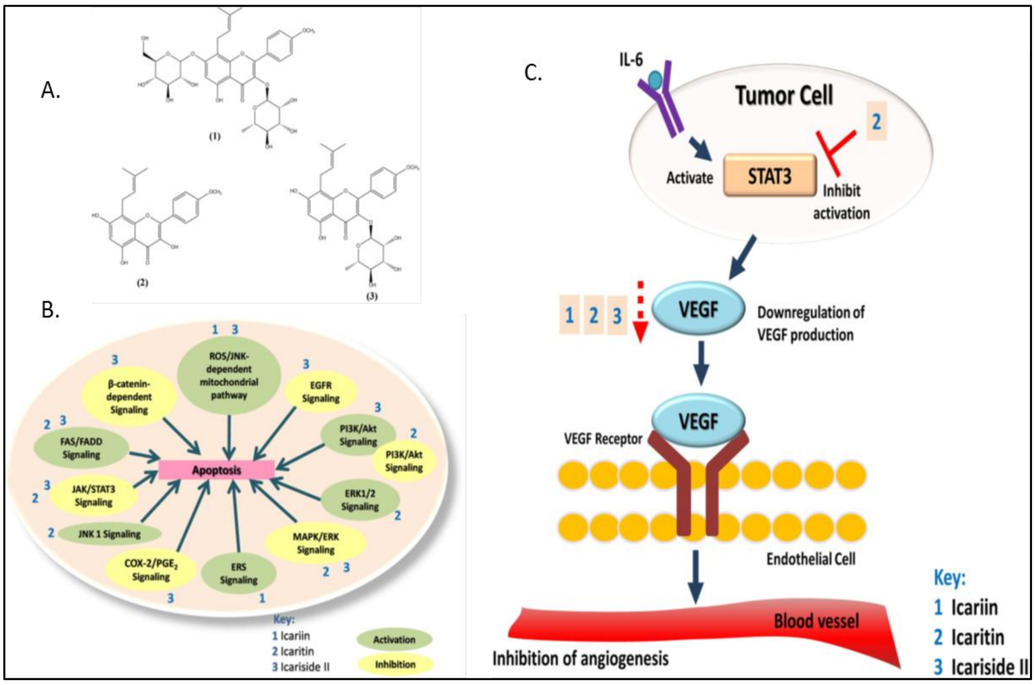

Fig.1S. Potential molecular basis of anti-cancer and immune-modulation targets/pathways associated with icaritin and its derivatives (modified from Tan HL et al. Front Pharmacol. 2016 Jun 29;7:191doi: 10.3389/fphar.2016.00191).

A. Chemical structures of icariin and its derivatives isolated from Herba Epimedii. Icariin (1), icaritin (2), andicariside II (3).

B. Icariin, icaritin, and icariside II exert apoptotic effects through multiple mechanisms, which include the inhibition of β-catenin-dependent signaling, EGFR signaling, MAPK/ERK signaling, PI3K/Akt signaling, JAK/STAT3 signaling, and COX-2/PGE2 signaling.

C. Potential targets and pathways of anti-Inflammation and anti-angiogenic effect by icaritin and its derivatives.
