# Supplementary material for: First-in-class immune-modulating small molecule Icaritin in advanced hepatocellular carcinoma: preliminary results of safety, durable survival and immune biomarkers
Source: BMC Cancer. 2019 Mar 28;19:279. doi: 10.1186/s12885-019-5471-1 (PMC6437929; doi:10.1186/s12885-019-5471-1)
Supplement: Supplementary file 2 — Figure S2. Diagram of adaptive phase I trial design. (PDF 113 kb) [file 12885_2019_5471_MOESM2_ESM.pdf]

Fig. 2S

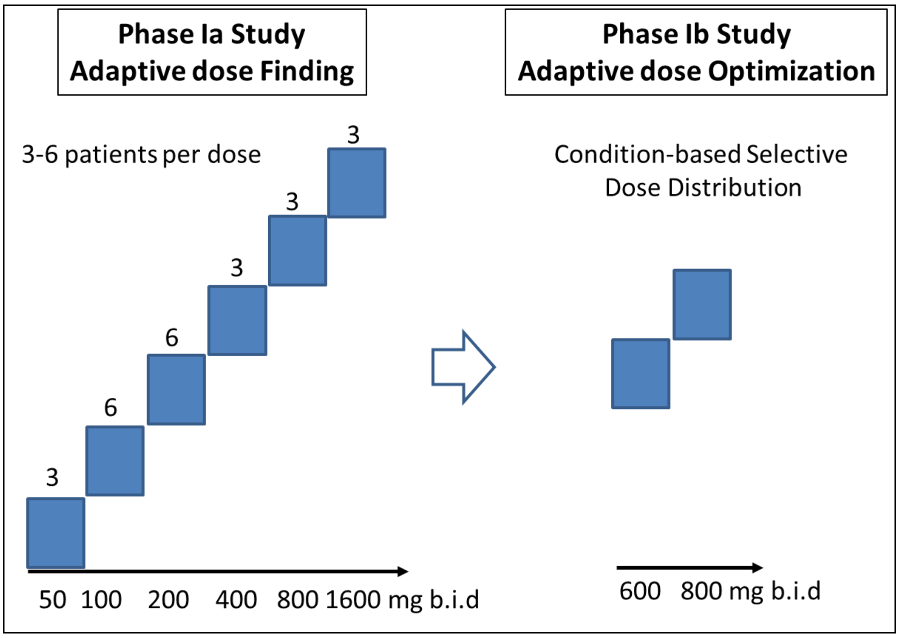

Fig. 2S. Diagram of adaptive phase I trial design; part 1 for dose escalation, and part 2 for dose optimization. Twenty-four patients with breast cancer were enrolled in part 1; There was no MDT dose observed in part 1. Part 2, Patients with advanced HCC (n=20); Breast cancer (n=1) and colorectal cancer (n=2), were enrolled and administrated with doses of icaritin 600 or 800 mg b.i.d .
