# Supplementary material for: First-in-class immune-modulating small molecule Icaritin in advanced hepatocellular carcinoma: preliminary results of safety, durable survival and immune biomarkers
Source: BMC Cancer. 2019 Mar 28;19:279. doi: 10.1186/s12885-019-5471-1 (PMC6437929; doi:10.1186/s12885-019-5471-1)
Supplement: Supplementary file 3 — Supplement methods for Fig. 3. (PDF 412 kb) [file 12885_2019_5471_MOESM3_ESM.pdf]

Supplement for methods of Fig. 3

Method A: Bone marrow-derived macrophages and icaritin treatment. Bone marrow-derived macrophages were differentiated with IL-4 and IL-13 for M2, or stimulated with LPS for positive control. qPCR was performed on bone marrow-derived macrophages (BMDMs). Relative copy numbers of M1- and M2-type genes were calculated based on β-actin. Bone marrow cells from Balb/c mice were cultured with M-CSF (10ng/ml) for 5 days, mediums were changed every two days. IL-4 and IL-13 (10ng/ml each) were added on day6 to induce M2-like BMDMs. Mediums were changed again on day7 with the indicated does of SNG162. After 24hrs, cells were collected and performed RNA extraction with Trizol, following the manufacturer's instruction. LPS (100ng/ml) was added at the last 6 hours as a positive control. First-strand cDNA was synthesized (NEB) and then subjected to qPCR (Bio-RadCFX96) with SYBR Green qPCR SuperMix (TransGen) using the corresponding primers. Gene expression was normalized to β-actin using the △△Ct method.

Method B. **Cord Blood-Derived Myeloid-Derived Suppressor Cells and DMSC treatment**

The method for expansion of $CD34^+$ progenitors was described in (ref). Briefly, $CD34^+$ cells were isolated from fresh human cord blood with CD34 MicroBead Kit (Miltenyi Biotec, Cat#130-046-702), according to the manufacturer's instructions. These $CD34^+$ cells were expanded for 7 days in StemSpan serum-free expansion medium (Stem Cell Technologies, Cat#09650) supplemented with 100 ng/mL SCF, 100 ng/mL FLT3L, 100 ng/mL TPO, and 20 ng/mL IL-3 (R&D Systems). Fresh expansion medium was changed every other day.

To obtain Cord blood-derived myeloid-derived suppressor cells, the expanded $CD34^+$ cells were plated at 2.5×10^5 per well in 24-well plates in complete DMEM medium supplemented with G-CSF (40 ng/mL) and IL-6 (40 ng/mL), then cultured at 37 ℃ in 5% CO_2-humidified atmosphere for 3–4 d(1). $CD34^+$ cells were incubated with icaritin (2.5μM, 72 hours) prior to cytokine stimulation.
