# Supplementary material for: First-in-class immune-modulating small molecule Icaritin in advanced hepatocellular carcinoma: preliminary results of safety, durable survival and immune biomarkers
Source: BMC Cancer. 2019 Mar 28;19:279. doi: 10.1186/s12885-019-5471-1 (PMC6437929; doi:10.1186/s12885-019-5471-1)
Supplement: Supplementary file 4 — Figure S4. Correlation analysis of overall survival (OS) association with immune dynamics and biomarkers. (PDF 154 kb) [file 12885_2019_5471_MOESM4_ESM.pdf]

Fig.4S

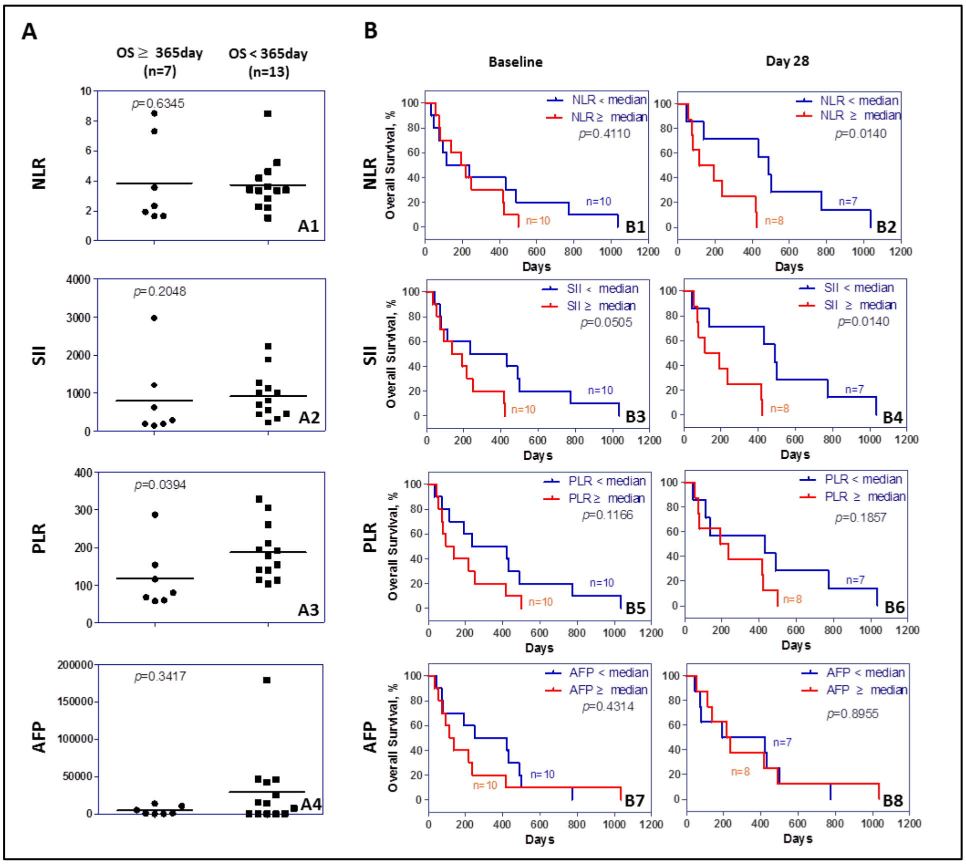

Fig. 4S. Correlation analysis of overall survival (OS) association with immune dynamics and biomarkers

(A) Biomarker difference between long- and short OS (365 day as cutoff) subgroups at the baseline. NLR: neutrophil to lymphocyte ratio; SII: Systemic Immune-Inflammation Index; PLR: platelet to lymphocyte ratio; AFP: alpha-fetoprotein.

(B) Kaplan-Meier curves for survival (OS) at baseline D0 and D28 of different immune biomarkers. B1, B3, B5 and B7 showed the relationships between OS and biomarkers at the baseline. The cutoff values were medians of 20 HCC patients for each factor. B2, B4, B6 and B8 show the relationships between OS and biomarkers after 1 cycle treatment (day 28). The cutoff values were medians of 15 HCC patients for each factor (rest of 5 patients were not evaluable).
