# Supplementary material for: First-in-class immune-modulating small molecule Icaritin in advanced hepatocellular carcinoma: preliminary results of safety, durable survival and immune biomarkers
Source: BMC Cancer. 2019 Mar 28;19:279. doi: 10.1186/s12885-019-5471-1 (PMC6437929; doi:10.1186/s12885-019-5471-1)
Supplement: Supplementary file 5 — Figure S5. Icaritin induced clinical activity and dynamic biomarkers. (PDF 181 kb) [file 12885_2019_5471_MOESM5_ESM.pdf]

Fig. 5S

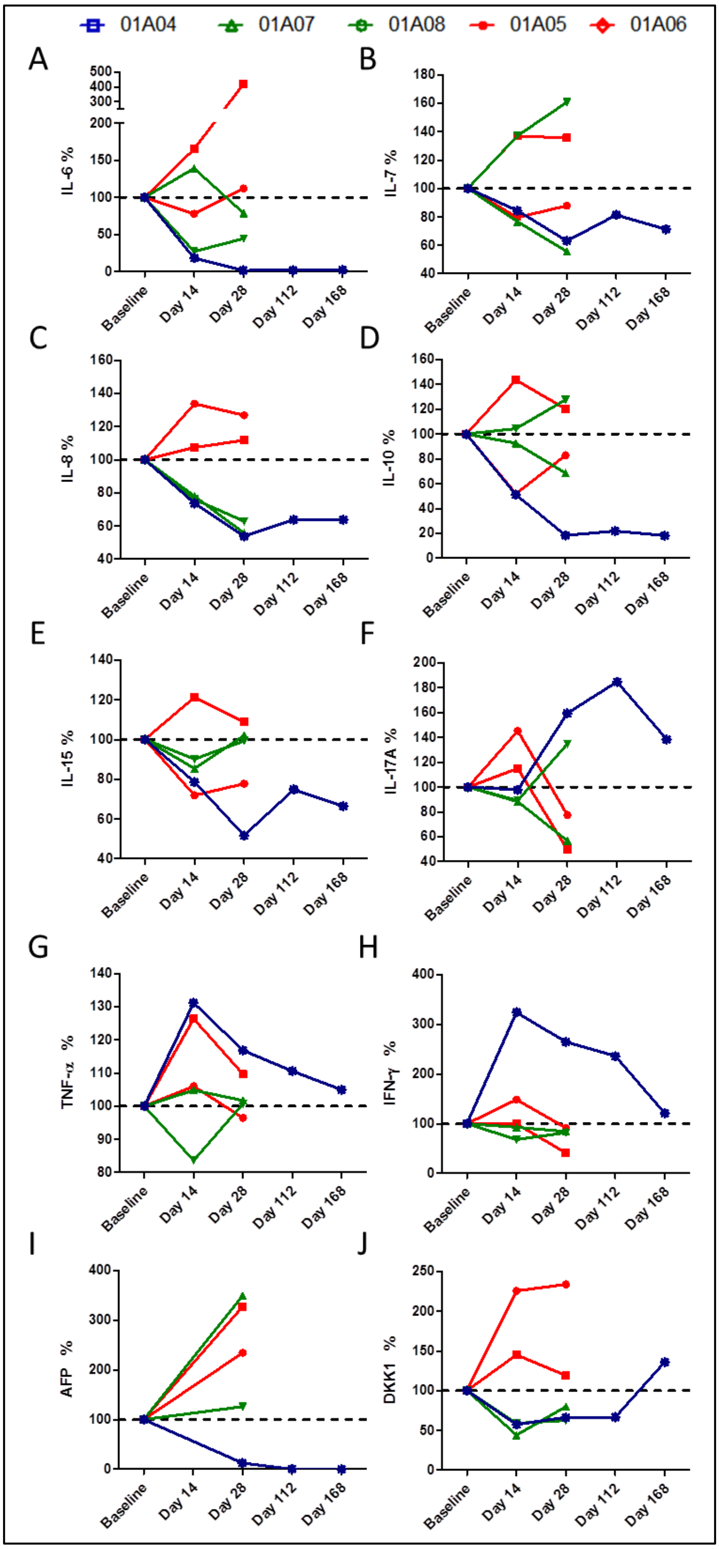

Fig.5S. Icaritin treatment induced clinical activity and dynamic biomarkers. HCC patients include patients of PR (01A04), SD (01A07, 01A08) and PD (01A05, 01A06). Methods: For DKK1, a commercial ELISA kit (R&D Systems, Minneapolis, MN, USA) was used according to the method of Shen et al. (The Lancet Oncology. 2012;13(8):817-26). The changes in cytokines such as IFN-γ, IL-2, IL-6, IL-8, IL-10, TNF-α, GM-CSF, IL-7, IL-15, and IL-17A were measured

with a customized platform on a Sector Imager 6000 instrument (MSD, Gaithersburg, MD, USA).
