# Supplementary material for: First-in-class immune-modulating small molecule Icaritin in advanced hepatocellular carcinoma: preliminary results of safety, durable survival and immune biomarkers
Source: BMC Cancer. 2019 Mar 28;19:279. doi: 10.1186/s12885-019-5471-1 (PMC6437929; doi:10.1186/s12885-019-5471-1)
Supplement: Supplementary file 6 — Table S1. Association between Dosage and Response. (PDF 248 kb) [file 12885_2019_5471_MOESM6_ESM.pdf]

**Supplement Table 1.** Association between dosage and response						
	Group					
Dosage	NE	PD	PD/NE	PR	SD	Total
600 mg bid	1	6	1	1	5	14
	5.00	30.00	5.00	5.00	5.00	70.00
	7.14	42.86	7.14	7.14	35.71	
	50.00	75.00	33.33	100.00	83.33	
800 mg bid	1	2	2	0	1	6
	5	10	10	0	5	30.00
	16.67	33.33	33.33	0	16.67	
	50	25	66.67	0	16.67	
Total	2	8	3	1	6	20
	10	40	15	5	30	100.00
		Fisher's Exact Test		Table probability (P)		0.0260
				Pr <=P		0.5489

Abbreviations: PR: Partial Response; SD: Stable Disease; PD: Progression Disease; NE: Non-RECIST evaluation;
