# Supplementary material for: First-in-class immune-modulating small molecule Icaritin in advanced hepatocellular carcinoma: preliminary results of safety, durable survival and immune biomarkers
Source: BMC Cancer. 2019 Mar 28;19:279. doi: 10.1186/s12885-019-5471-1 (PMC6437929; doi:10.1186/s12885-019-5471-1)
Supplement: Supplementary file 7 — Figure S3. Association between dosage and mortality. (PDF 106 kb) [file 12885_2019_5471_MOESM7_ESM.pdf]

**Fig.3S**

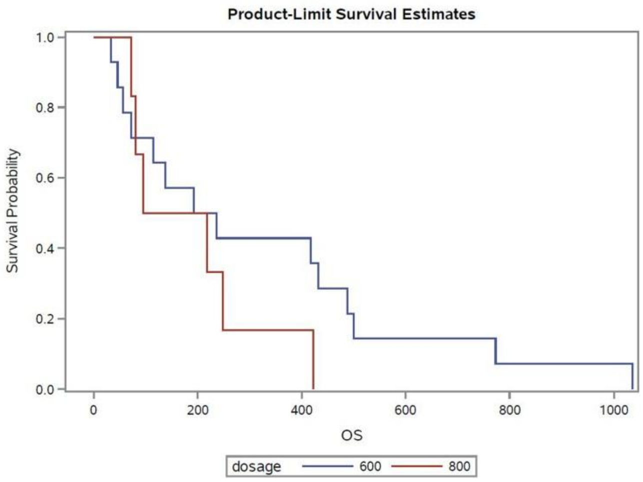


**Fig.3S**. Association between dosage and mortality. P value for log rank test=0.2752; p-value for Cox regression = 0.2811 (HR=1.778, 95%CI: [0.624, 5.064]).
