# Supplementary material for: First-in-class immune-modulating small molecule Icaritin in advanced hepatocellular carcinoma: preliminary results of safety, durable survival and immune biomarkers
Source: BMC Cancer. 2019 Mar 28;19:279. doi: 10.1186/s12885-019-5471-1 (PMC6437929; doi:10.1186/s12885-019-5471-1)
Supplement: Supplementary file 8 — Table S2. Baseline Characteristics and survival of HCC Patients (Comparison with the Chinese patients of Oriental Trial). (PDF 277 kb) [file 12885_2019_5471_MOESM8_ESM.pdf]

Supplement Table 2. Baseline characteristics and survival of HCC patients

Characteristic	Icaritin 600 mg bid (N=14)	Icaritin 800mg bid (N=6)	Icaritin Total (N=20)	Oriental Trial Sorafenib (N=150)	Oriental Trial Placebo (N=76)
Median age, years (range)	61 (31-74)	43 (33-73)	58 (31-74)	51 (23−86)	52 (25−79)
Male, no. (%)	12 (85.7)	5 (83.3)	17 (85.0)	127 (84.7)	66 (86.8)
ECOG performance status, no. (%)					
0	2 (14.3)	0 (0.0)	2 (10.0)	38 (25.3)	21 (27.6)
1	11 (78.6)	6 (100.0)	17 (85.0)	104 (69.3)	51 (67.1)
2	1 (7.1)	0 (0.0)	1 (5.0)	8 (5.3)	4 (5.3)
Macroscopic vascular invasion, no. (%)					
Yes	5 (35.7)	2 (33.3)	7 (35.0)	54 (36.0)	26 (34.2)
No	9 (64.3)	4 (66.7)	13 (65.0)	96 (64.0)	50 (65.8)
Portal vein tumor thrombus, no. (%)					
Yes	3 (21.4)	2 (33.3)	5 (25.0)	---	---
No	11 (78.6)	4 (66.7)	15 (75.0)	---	---
Bulky tumor*, no. (%)					
Yes	1 (7.1)	1 (16.7)	2 (10.0)	---	---
No	13 (92.9)	5 (83.3)	18(90.0)	---	---
Extrahepatic metastasis, no. (%)					
Yes	13 (92.9)	5 (83.3)	18 (90.0)	103 (68.7)	52 (68.4)
No	1 (7.1)	1 (16.7)	2 (10.0)	47 (31.3)	24 (31.6)
Extrahepatic metastatic site, no. (%)					
Lymph node	7 (50.0)	4 (66.7)	11 (55.0)	46 (30.7)	26 (34.2)
Lung	7 (50.0)	3 (50.0)	10 (50.0)	78 (52.0)	34 (44.7)
BCLC stage, no. (%)					
B	1 (7.1)	0 (0.0)	1 (5.0)	---	---
C	13 (92.9)	6 (100.0)	19 (95.0)	143 (95.3)	73 (96.1)
Number of tumor sites, no. (%)					
1	2 (14.3)	1 (16.7)	3 (15.0)	20 (13.3)	5 (6.6)
2	6 (42.9)	1 (16.7)	7 (35.0)	52 (34.7)	27 (35.5)
3	3 (21.4)	2 (33.3)	5 (25.0)	30 (20.0)	14 (18.4)
≥4	3 (21.4)	2 (33.3)	5 (25.0)	48 (32.0)	30 (39.5)
Hepatitis virus status, no. (%)					
HBV infection	12(85.7)	6 (100.0)	18 (90.0)	106 (70.7)	59 (77.6)
HCV infection	1 (7.1)	0 (0.0)	1 (5.0)	16 (10.7)	3 (3.9)
Liver cirrhosis, no. (%)					
Yes	10 (71.4)	5 (83.3)	15 (75.0)	---	---
No	4 (28.6)	1 (16.7)	5 (25.0)	---	---
Child-Pugh classification, no. (%)					
A	13 (92.9)	5 (83.3)	18 (90.0)	146 (97.3)	74 (97.4)

B	1 (7.1)	1 (16.7)	2 (10.0)	4 (2.7)	2 (2.6)
AFP > ULN (laboratory), no. (%)	13 (92.9)	6 (100.0)	19 (95.0)	116 (77.3)	59 (77.6)
Previous treatment, no. (%)					
Surgery	3 (21.4)	4 (66.7)	7 (35.0)	49 (32.7)	21 (27.6)
TACE/RFA	11 (78.6)	3 (50.0)	14 (70.0)	61 (40.7)	33 (43.4)
Sorafenib or Chemo	1 (7.1)	3 (50.0)	4 (20.0)	0	0
Survival (Days)					
Overall Survival (medium OS)	214	156.5	205	174*	114*
PR/SD Patients (medium OS)	460	422	432	---	---
PD Patients ((medium OS)	125.5	95	114	---	---

(* Oriental trial HCC patient from China, Ref. European J. Cancer, 2012, 48: 1452)
